# Correction: Characterizing organisms from three domains of life with universal primers from throughout the global ocean

**DOI:** 10.1038/s41597-026-08484-6

**Published:** 2026-10-22

**Authors:** Jesse McNichol, Nathan L. R. Williams, Yubin Raut, Craig Carlson, Elisa R. Halewood, Kendra Turk-Kubo, Jonathan P. Zehr, Andrew P. Rees, Glen Tarran, Mary R. Gradoville, Matthias Wietz, Christina Bienhold, Katja Metfies, Sinhué Torres-Valdés, Thomas Mock, Sarah Lena Eggers, Wade Jeffrey, Joseph Moss, Paul Berube, Steven Biller, Levente Bodrossy, Jodie Van De Kamp, Mark Brown, Swan L. S. Sow, E. Virginia Armbrust, Jed Fuhrman

**Affiliations:** 1 Department of Biological Sciences–Marine and Environmental Biology, University of Southern California, Los Angeles, California USA; 2 Department of Biology, St. Francis Xavier University, Antigonish, Nova Scotia Canada; 3 Ecology, Evolution, and Marine Biology, University of California, Santa Barbara, CA USA; 4 Ocean Sciences Department, University of California Santa Cruz, Santa Cruz, CA USA; 5 Plymouth Marine Laboratory, Plymouth, UK; 6 Columbia River Inter-Tribal Fish Commission, Portland, Oregon USA; 7 Alfred Wegener Institute Helmholtz Centre for Polar and Marine Research, Bremerhaven, Germany; 8 Max Planck Institute for Marine Microbiology, Bremen, Germany; 9 School of Environmental Sciences, University of East Anglia, Norwich Research Park, UK; 10 Center for Environmental Diagnostics and Bioremediation, University of West Florida, Pensacola, Florida USA; 11 Department of Civil and Environmental Engineering, Massachusetts Institute of Technology, Cambridge, Massachusetts USA; 12 Department of Biological Sciences, Wellesley College, Wellesley, Massachusetts USA; 13 CSIRO Environment, Hobart, Tasmania Australia; 14 Independent Researcher, Hobart, Tasmania Australia; 15 Nantes Université, École Centrale Nantes, Nantes, France; 16 School of Oceanography, University of Washington, Seattle, WA USA

Correction to: *Scientific Data* https://doi.org/10.1038/s41597-025-05423-9, published online 01 July 2025

“In this article, the sentence “AMT19/20: PRJNA1133034” is missing from the last paragraph of the Methods section, under the subsection heading “Sequencing methods and pipeline”:

“The forward and reverse sequences were submitted to the NCBI database under accession numbers GA02/ GA10: PRJNA1194189, GA03: PRJNA1194192, GP13: PRJNA1195113, Scope-Gradients 2: PRJNA1195115, Scope-Gradients 3: PRJNA1196422, P16N: PRJNA1196483, P16S: PRJNA1196490, P15S: PRJNA1196498, HEOBI: PRJNA1196504, IND-2017: PRJNA1196513, K-AXIS: PRJNA119651, POTATOE: PRJNA1198072, I08S: PRJNA1198088, I09N: PRJNA1198607, MOSAiC: PRJNA1198992, and FRAM: PRJNA1199044. The scripts necessary to exactly reproduce the analysis are available at https://github.com/jcmcnch/ Global-rRNA-Univeral-Metabarcoding-of-Plankton, with modifications to the merge script found at https:// github.com/Nwilliams96/Project-2-Universal-Primer-Pipeline-Modifications”.

In Table 3 Cruise bioGEOTRACES GP13 “Ocean” should be the “Pacific Ocean” the original article has been corrected”.

