# Supplementary material for: Characterizing organisms from three domains of life with universal primers from throughout the global ocean
Source: Sci Data. 2025 Jul 1;12:1078. doi: 10.1038/s41597-025-05423-9 (PMC12218243; doi:10.1038/s41597-025-05423-9)
Supplement: Supplementary file 1 — Full Supplementary Material [file 41597_2025_5423_MOESM1_ESM.docx]

**Characterizing organisms from three domains of life with universal primers from throughout the global ocean**

Jesse McNichol^a,b†^, Nathan L R Williams^a†^, Yubin Raut^a^, Craig Carlson^c^, Elisa R Halewood^c^, Kendra Turk-Kubo^d^, Jonathan P Zehr^d^, Andrew P Rees^e^, Glen Tarran^e^, Mary R. Gradoville^f^, Matthias Wietz^g,h^, Christina Bienhold^g,h^, Katja Metfies^g^, Sinhué Torres-Valdés^g^, Thomas Mock^i^, Sarah Lena Eggers^g^, Wade Jeffrey^j^, Joseph Moss^j^, Paul Berube^k^, Steven Biller^l^, Levente Bodrossy^m^, Jodie Van De Kamp^m^, Mark Brown^p^, Swan L. S. Sow^n^, E. Virginia Armbrust^o^, Jed Fuhrman^a^

^†^Co-first authors.

^a^ Department of Biological Sciences–Marine and Environmental Biology, University of Southern California, Los Angeles, California, United States

^b^ Department of Biology, St. Francis Xavier University, Antigonish, Nova Scotia, Canada

^c^ Ecology, Evolution, and Marine Biology, University of California, Santa Barbara, CA, United States

^d^ Ocean Sciences Department, University of California Santa Cruz, Santa Cruz, CA, United States

^e^ Plymouth Marine Laboratory, Plymouth, United Kingdom

^f^ Columbia River Inter-Tribal Fish Commission, Portland, Oregon, United States

^g^ Alfred Wegener Institute Helmholtz Centre for Polar and Marine Research, Bremerhaven, Germany

^h^ Max Planck Institute for Marine Microbiology, Bremen, Germany

^i^ School of Environmental Sciences, University of East Anglia, Norwich Research Park, United Kingdom

^j^ Center for Environmental Diagnostics and Bioremediation, University of West Florida, Pensacola, Florida, United States

^k^ Department of Civil and Environmental Engineering, Massachusetts Institute of Technology, Cambridge, Massachusetts, United States

^l^ Department of Biological Sciences, Wellesley College, Wellesley, Massachusetts, United States

^m^ CSIRO Environment, Hobart, Tasmania, Australia

^n^ Nantes Université, École Centrale Nantes, Nantes, France

^o^ School of Oceanography, University of Washington, Seattle, WA, United States

^p^ Independent Researcher, Hobart, Tasmania, Australia

**Supplementary Figure 1.** Map of FRAM sampling locations.


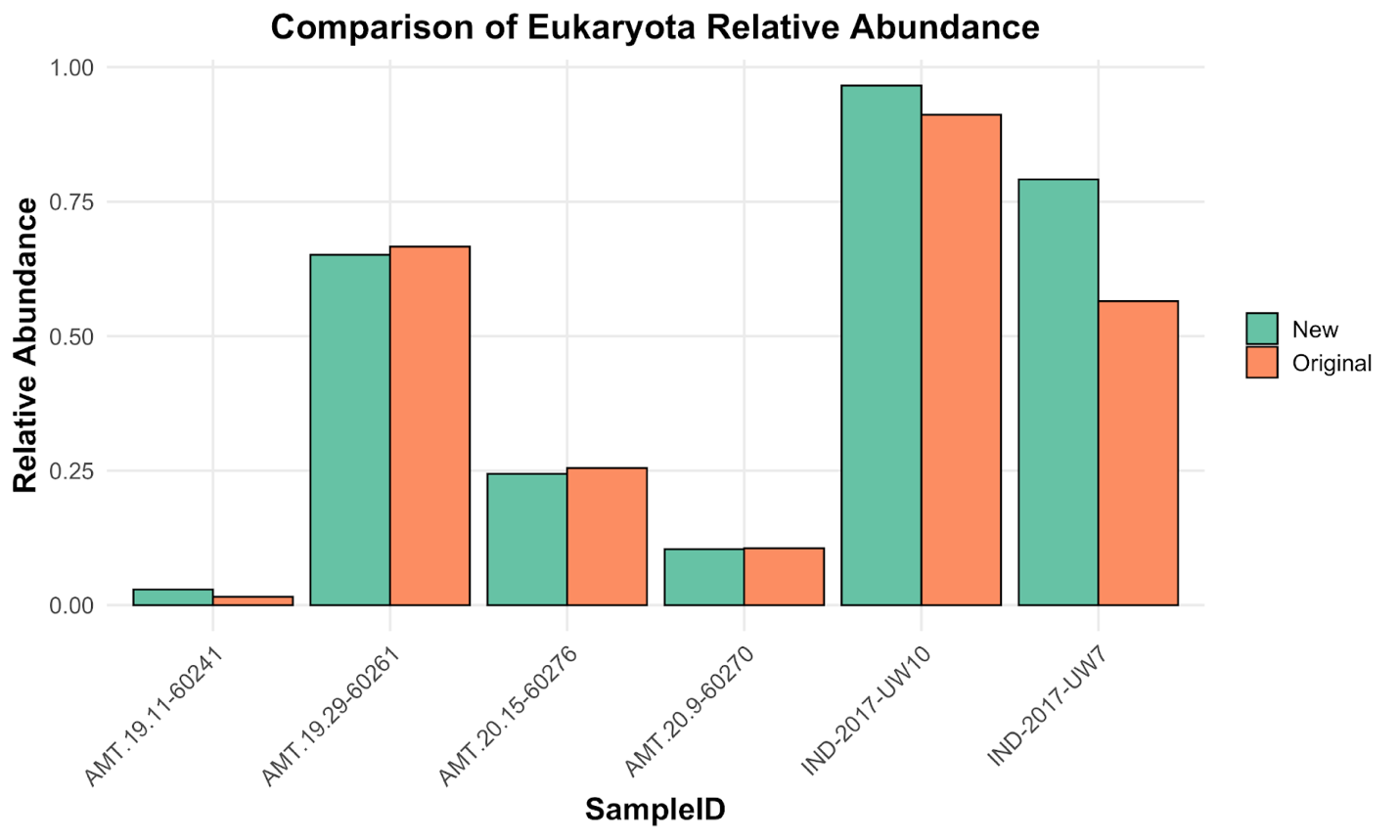


**Supplementary Figure 2**. Bar plot comparing re-sequenced Eukaryotic relative abundance compared to original sequencing. Note that the original samples from AMT had an original correction factor of 9.42 and new correction factor of 5.96 while the IND 2017 samples had an original correction factor of 1.90 and a new correction factor of 5.96. The re-run samples are in green and the original samples are in orange.


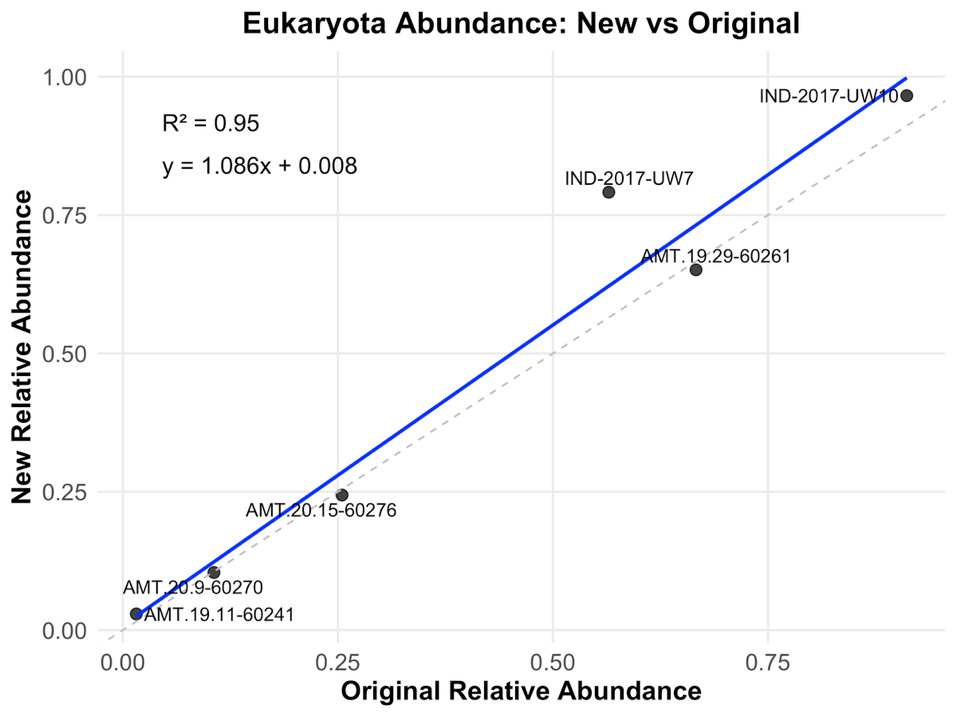


**Supplementary Figure 3**. Regression of the re-sequenced Eukaryotic relative abundance vs the original Eukaryotic Relative abundance, showing very close agreement in original and resequenced samples. The dashed line is a perfect 1:1 relationship.

B

A

**Supplementary Figure 4.** Relative abundance of marine microbes from the GA02 transect from the surface – 200 m of the ocean, and 200 m - 1000 m, partitioned between Archaea, Bacteria, Cyanobacteria, Chloroplast 16S, and Eukaryotic 18S. Relative abundance (y axis) is shown at increasing latitudes (x axis) from within the Atlantic Ocean (Cruise GA02). A is top 200m, B is 200-1000m depths.

C

B

A

**Supplementary Figure 5.** Relative abundance of marine microbes from the P16N cruise between the surface – 200 m of the ocean, 200 m - 1000 m, and then below 1000 m, partitioned between Archaea, Bacteria, Cyanobacteria, Chloroplast 16S, and Eukaryotic 18S. Relative abundance (y axis) is shown at increasing latitudes (x axis) from within the Pacific Ocean (Cruise P16N and P16S). A is top 200m, B is 200-1000m, C is below 1000m.

B

A

C

**Supplementary Figure 6.** Relative abundance of marine microbes from the P15S transect between the surface – 200 m of the ocean, 200 m - 1000 m, and then below 1000 m, partitioned between Archaea, Bacteria, Cyanobacteria, Chloroplast 16S, and Eukaryotic 18S. Relative abundance (y axis) is shown at increasing latitudes (x axis) from within the Southern Ocean (Cruise P15S). A is top 200m, B is 200-1000m, C is below 1000m.

**Supplementary Figure 7.** Relative abundance of marine microbes from the I9N and I8S transects between the surface – 200 m of the ocean, 200 m - 1000 m, and then below 1000 m partitioned between Archaea, Bacteria, Cyanobacteria, Chloroplast 16S, and Eukaryotic 18S. Relative abundance (y axis) is shown at increasing latitudes (x axis) from within the Indian Ocean (Cruise IO9N and IO8S). A is top 200m, B is 200-1000m, C is below 1000m.

C

B

A
